# Measles Vaccine Coverage and Disease Outbreaks: A Systematic Review of the Early Impact of COVID-19 in Low and Lower-Middle Income Countries

**DOI:** 10.3389/ijph.2024.1606997

**Published:** 2024-04-25

**Authors:** Alice Packham, Alice E. Taylor, Marie-Paule Karangwa, Emma Sherry, Claude Muvunyi, Christopher A. Green

**Affiliations:** ^1^ School of Chemical Engineering, College of Engineering and Physical Sciences, University of Birmingham, Birmingham, England, United Kingdom; ^2^ Department of Infectious Diseases and Tropical Medicine, University Hospitals Birmingham NHS Foundation Trust, Birmingham, United Kingdom; ^3^ Rwanda Biomedical Center, Kigali, Rwanda

**Keywords:** COVID-19, measles, vaccine coverage, immunization agenda 2030, inequalities of health

## Abstract

**Objectives:** We aimed to evaluate changes to measles-containing vaccine (MCV) provision and subsequent measles disease cases in low- and lower-middle income countries (LICs, LMICs) in relation to the COVID-19 pandemic.

**Methods:** A systematic search was conducted of MEDLINE, OVID EMBASE and PubMed records. Primary quantitative and qualitative research studies published from January 2020 were included if they reported on COVID-19 impact on MCV provision and/or measles outbreak rates within LICs and LMICs.

**Results:** 45 studies were included. The change in MCV1 vaccination coverage in national and international regions ranged −13% to +44.4% from pre-COVID time periods. In local regions, the median MCV1 and overall EPI rate changed by −23.3% and −28.5% respectively. Median MCV2 rate was disproportionally impacted in local areas during COVID-interruption time-periods (−48.2%) with ongoing disruption in early-recovery time-periods (−17.7%). 8.9% of studies reported on vaccination status of confirmed measles cases; from these, 71%–91% had received no MCV dose.

**Conclusion:** MCV vaccination coverage experienced ongoing disruption during the recovery periods after initial COVID-19 disruption. Vaccination in local area datasets notably experienced longer-term disruption compared to nationally reported figures.

## Introduction

Measles paramyxovirus is a human-restricted viral pathogen without environmental or animal reservoirs. Infection and/or vaccination induces durable immune protection from developing severe disease and can restrict onward transmission. This supports the prospect of measles eradication through public health programmes of surveillance and vaccination, and the elimination of measles in at least five WHO regions as a major priority of the United Nations Children’s Fund (UNICEF) [1]. The WHO Immunization Agenda 2021–2030 defines the threshold of 95% measles vaccination coverage to achieve herd immunity, by using measles-containing vaccines (MCV) as part of the WHO’s expanded programme on immunisation (EPI) schedule for infants since 1974. The regimen was revised to include a second MCV dose from 2009 [2, 3]. Immunity gaps to measles are often rapidly apparent due to the high infectivity of the virus with large and rapid disease outbreaks (the estimated basic reproduction number R₀ is 12–18).

Global MCV coverage has stagnated since 2010 to between 84% and 86% of the population [4]. 2019 marked the highest number of measles-related deaths in nearly two decades, with endemic transmission re-established in multiple countries which had previously sustained measles elimination targets [5]. The most challenging outbreaks occur in resource-deprived settings producing serious ramifications, as recently highlighted in the Democratic Republic of the Congo. Here, reduced measles vaccination coverage during the 2018–2020 Ebola outbreak contributed to a measles disease outbreak resulting in approximately 7,000 measles-related deaths, compared to 2,243 deaths from Ebola [6].

The COVID-19 pandemic further impaired sustained childhood immunisation coverage. WHO estimated that 22.7 million children missed routine immunizations in 2020, a 19.5% increase from 2019 [7]. UNICEF reported 67 million children were under-vaccinated or fully unvaccinated between 2019 and 2021, with a 5% decline in the number of children receiving their first dose of MCV (MCV1) [8]. In 2022, Cardoso Pinto et al published a systematic review demonstrating a median decline in routine paediatric vaccination of 10.8% during the early COVID-19 pandemic [9]. Vaccination coverage is disproportionately lower in lower-income countries. A 2021 worldwide vaccination report estimated that from 18.2 million unvaccinated children, 12.8 million (70%) lived in middle-income countries followed by five million (27%) in low-income countries (LICs) [10]. The WHO described the current setting as a “perfect storm” for measles outbreaks [11]. Recovering measles vaccination coverage to pre-pandemic levels is a United Nations urgent global priority and key in meeting the third sustainable development goal (SDG3: ensure health lives and promote wellbeing for all at all ages) [12]. Expanding vaccination services to reach these children and reducing vaccine inequity is a strategic priority of the 2030 Immunisation Agenda [3] alongside strengthening capacities for responsiveness to outbreaks. This aims to support the Gavi strategy (5.0) for equitable vaccination coverage, with a 25% reduction in the number of zero-dose children by 2025 through reaching missed communities [13]. The extent to which COVID-19 has de-railed such targets remains to be seen.

Measles can be considered the “canary in the immunological coalmine” for all childhood immunisations [14] and is useful for looking at the scale and long-term implications of disrupted childhood vaccination coverage [15]. We present the following systematic review on the impact of the COVID-19 pandemic on MCV coverage and outbreaks in LICs and lower-middle-income countries (LMICs). To our knowledge, no such systematic review has been published previously. We aim to highlight the impacts of COVID-19 disruptions on global vaccine supply chains as well as the importance of building resilience to inevitable future pandemics and evolving challenges to ensure progression towards achieving development goals.

## Methods

### Registration

Our methods adhere to the Preferred Reporting Items for Systematic Reviews and Meta-Analyses (PRISMA) [16] (Supplementary Appendix S1). This systematic review was prospectively registered on Prospero (ID: CRD42023394215).

### Search Strategy

The search was conducted on three databases on 26 January 2023: OVID Medline, OVID Embase and PubMed. The search was limited to publications from January 2020 onwards. No limitations were placed on language. The search strategy included three themes: measles, immunisation programmes, and low-income countries, using proximity Boolean operators (Supplementary Appendix S2). No limitations were placed on COVID-19.

### Study Selection

Primary research studies reporting on measles vaccine rate and coverage before, during, and after the COVID-19 pandemic, either alone or as part of wider childhood immunisation data, were included. Quantitative, qualitative, and primary research studies with data on measles outbreaks, measles vaccination, and the perception of measles vaccination before and after COVID-19 disruption were included.

Studies were restricted to data from LIC and LMIC settings. Any countries that changed status during the period of study were included if they were classed as LIC/LMIC at any time during data collection for the publication. Where studies used data derived from multiple countries, individual LICs or LMICs were included if there was clear discussion of country-specific data. WHO regions were included where ³75% of countries within the region were classed as LIC/LMIC during 2020. This included the Southeast Asia region and Africa region only (Supplementary Appendix S3). Age range was not limited.

Studies with sole focus on COVID-19 vaccination and modelling studies were excluded. Editorials, opinion pieces, news articles, reviews without empirical data and non-peer reviewed articles were excluded. Language restrictions were not applied; non-English language publications were translated to English using Google Translate, and studies were excluded if this translation was unclear. EndNote (version 20.5, Clarivate) and Microsoft Excel 365 (version 2,308, Microsoft) were used to remove duplicates. Abstract and full text screening was completed on Microsoft Excel 365 by two independent investigators. If discrepancies were not resolved between two investigators, a third investigator was consulted as arbitrator.

### Data Collection, Synthesis, and Presentation

Data was collected by two independent investigators. A pre-prepared, standardised, data extraction sheet (Microsoft Excel 365 (version 2308, Microsoft)) collected outcomes of interest that included reported changes in vaccination (by rate or coverage) for measles-containing vaccines and/or EPI programmes during and after COVID-19-related disruptions in 2020; measles disease outbreaks from March 2020 onwards; and reasons discussed for changes in vaccine provision and/or measles cases rates. Data included the reported measles vaccination coverage, full and incomplete, before and after the WHO declaration date of the COVID-19 pandemic on 11 March 2020. This included any measles-containing vaccines used in isolation or as part of EPI or supplementary immunization activities (SIA), as well as important metadata such as geographic, demographic, and socio-economic context. Quantitative data was grouped into changes in rate or coverage for measles-containing vaccines or EPI vaccines for analysis. Qualitative data was grouped by characteristic and displayed as a table. Analysis and figure generation was performed using GraphPad Prism (GraphPad, version 9.5.1, 528) and sheet Microsoft Excel 365 (version 2308, Microsoft).

### Quality Assessment

Quality assessment was carried out using the National Institute of Health Quality Assessment Tool for Observational Cohort and Cross-Sectional Studies (NIHQAT) [17] for quantitative studies and the Critical Appraisal Skills Programme (CASP) [18] for qualitative studies. Assessment of each included study was carried out by two independent investigators with a third investigator consulted for any discrepancies.

## Results

**FIGURE 1:**
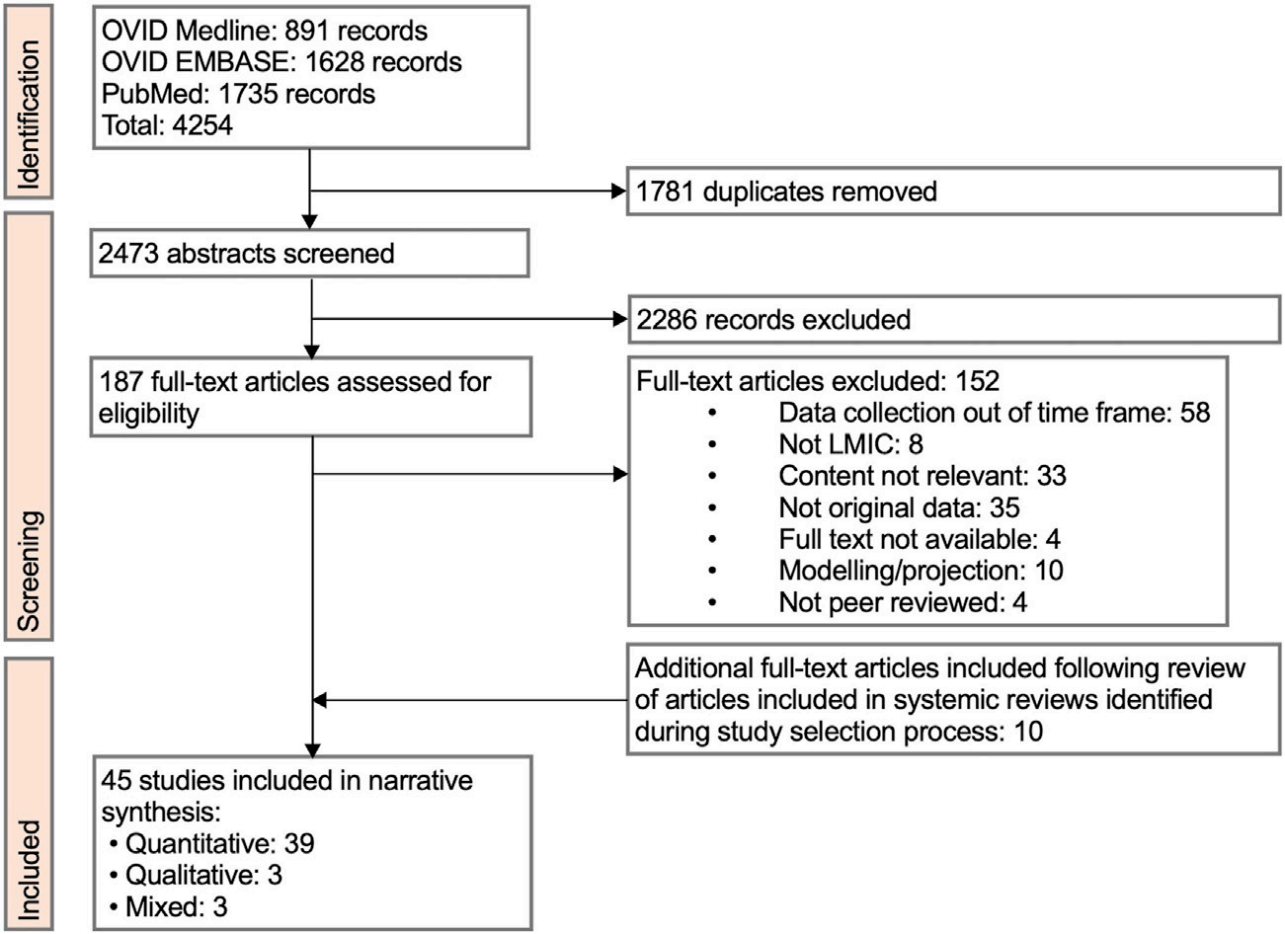
Preferred Reporting Items for Systematic Reviews and Meta-Analyses (PRISMA) flow diagram of review study selection (Low-income countries and lower-middle income countries, 2023).

4,254 studies were identified through the initial literature search (Figure 1). 1781 duplicates were removed. 2,473 abstracts were screened. 187 full-text articles were assessed for eligibility. 152 full texts were excluded. 10 additional studies were included during full-text review from analysis of references. In total, 45 studies were included in the review.

45 eligible studies collectively reported data from 112 areas within LIC/LMIC countries, regions or WHO regions (Table 1, descriptive summary of studies in Supplementary Appendix S4). 92/112 (82%) were in the Africa (AFR) region, 17/112 (15%) were in South East Asia (SEA) region and 3/112 (3%) were from other WHO regions. The countries most frequently represented were Ethiopia (*n* = 6), India (*n* = 6) and Nigeria (*n* = 6). The proportion of studies assessing LIC, LMIC or a combination of LIC and LMIC was *n* = 11 (24%), *n* = 25 (56%), and *n* = 9 (20%) respectively. 26/45 (58%) of studies assessed data from local regions within country.

**TABLE 1:** Summary of included studies (Low-income countries and lower-middle income countries, 2020–2023).

Locations investigated in included studies						Details of 45 included papers	
Country	No. of studies (% of total)	Study references	Country	No. of studies (% of total)	Study references	Characteristic	No. of papers (% of total)
**Africa (AFR)**			**Africa (AFR) cont.**			**Income level**	
			Senegal	2 (1.8)	[19, 20]	LIC	11 (22.4)
AFR region	5 (4.4)	[21–25]	Sierra Leone	2 (1.8)	[19, 26]	LMIC	25 (55.6)
Algeria	1 (0.9)	[19]	South Sudan	3 (2.6)	[19, 20, 25]	LIC and LMIC	9 (20.0)
Angola	2 (1.8)	[19, 20]	Tanzania	3 (2.6)	[19, 20, 25]		
Benin	1 (0.9)	[19]	Togo	1 (0.9)	[19]	**Scope of data**	
Burundi	2 (1.8)	[19, 20]	Uganda	4 (3.5)	[19, 25, 27, 28]	Global	3 (6.7)
Cameroon	2 (1.8)	[19, 29]	Zambia	2 (1.8)	[19, 25]	Multinational	6 (13.3)
CAR	2 (1.8)	[19, 20]	Zimbabwe	3 (2.6)	[19, 25]	National	10 (22.2)
Chad	2 (1.8)	[19, 20]		**92 (80.7)**		Local	26 (57.8)
Cape Verde	1 (0.9)	[19]					
DRC	2 (1.8)	[19, 20]	**South-East Asia (SEA)**			**Data source**	
Eritrea	3 (2.6)	[19, 20, 25]	SEA region	4 (3.5)	[21–24]	Health records/database (govt or local authority)	14 (31.1)
Eswatini	2 (1.8)	[19, 25]	Bhutan	1 (0.9)	[30]		
Ethiopia	6 (5.3)	[19, 25, 31–34]	India	6 (5.3)	[35–40]	Health records/database (hospital/medical center)	12 (26.7)
Gambia	2 (1.8)	[19, 41]	Nepal	1 (0.9)	[42]		
Ghana	4 (3.5)	[19, 20, 43, 44]	Pakistan	4 (3.5)	[45–48]	WHO data source	4 (8.9)
Guinea	2 (1.8)	[19, 20]	Viet Nam	1 (0.9)	[49]	NGO records	0 (0.0)
Guinea-Bissau	1 (0.9)	[19]		**17 (14.9)**		Surveys	1 (2.2)
Kenya	5 (4.4)	[19, 20, 25, 50, 51]	**Americas**			Literature searches	1 (2.2)
Lesotho	3 (2.6)	[19, 25, 52]	Haiti	1 (0.9)	[52]	Interview/questionnaires	5 (11.1)
Liberia	1 (0.9)	[19]		**1 (0.9)**		Not clear	3 (6.7)
Madagascar	2 (1.8)	[19, 25]				Multiple*	5 (11.1)
Malawi	3 (2.6)	[19, 25, 52]	**European**				
Mali	2 (1.8)	[19, 25]	Ukraine	1 (0.9)	[53]	**Time periods**	
Mauritania	1 (0.9)	[19]		**1 (0.9)**		2019 to 2020	15 (33.3)
Mozambique	3 (2.6)	[19, 25, 54]	**Eastern Mediterranean (EM)**			2019 to 2021	8 (17.8)
Niger	1 (0.9)	[19]	Afghanistan	1 (0.9)	[55]	2020 (early) to 2020 (late)	9 (20.0)
Nigeria	6 (5.3)	[19, 20, 56–59]		**1 (0.9)**		2020 to post-2020	3 (6.7)
Rwanda	4 (3.5)	[19, 20, 25, 60]	**Global**	2 (1.8)	[10, 61]	Pre-2019 to 2020 onwards	5 (11.1)
Sao Tome and Principe	1 (0.9)	[19]		**2 (1.8)**		Other	5 (11.1)
			**Total**	**114 (100)**		**Type of study**	
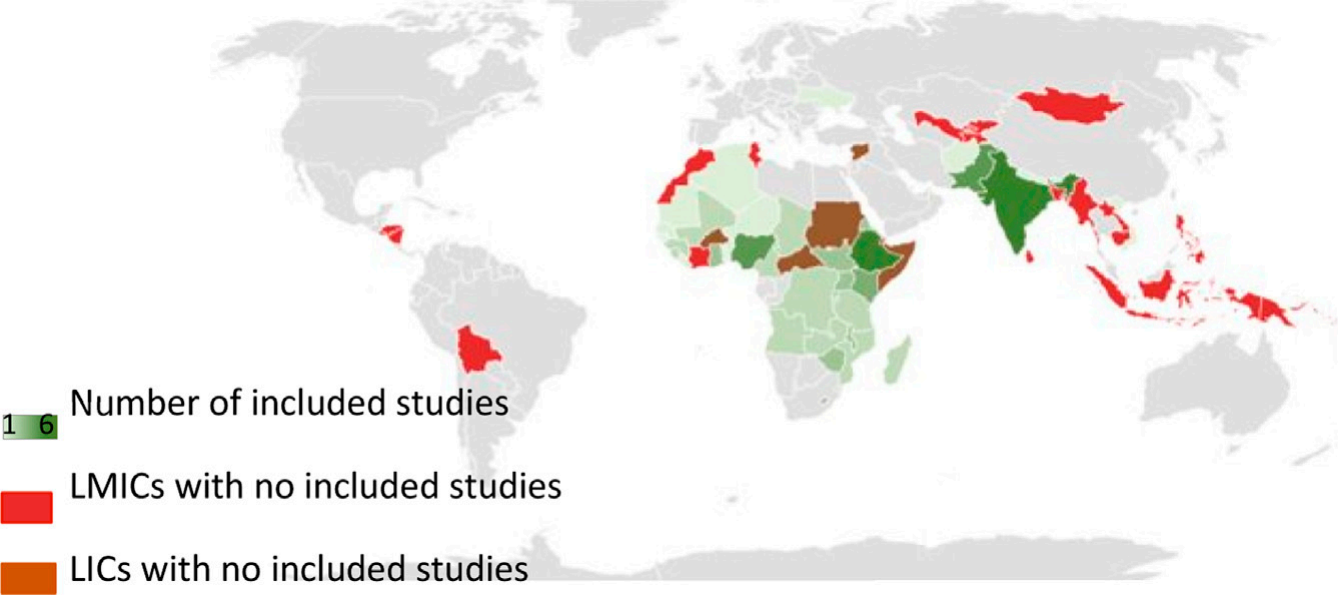						Quantitative	39 (86.7)
						Qualitative	3 (6.7)
						Mixed	3 (6.7)
						**Focus of study**	
						Vaccination (rate and/or coverage)	34 (75.6)
						Outbreak	4 (8.9)
						Vaccination and outbreak	7 (15.6)
						**Global heat-map:** number of studies identified per LMIC/LIC during the systematic review and the LICs/LMICs for which no studies were identified	

^a^ Multiple sources: Atim et al [26], health records/database (govt or local authority) AND literature search; Bimpong et al [47] and Kissi et al [48], health records/database (at hospital or medical centre level) AND interview/questions; Nomhwange et al [56], health records/database (at hospital or medical centre level) AND interview/questionnaires AND Health records/database (govt or local authority); Barasa et al [50]; Interview/questionnaires AND Health records/database (govt or local authority).

Each bold figure is the running total per region, with percentage of total in brackets. This can be represented as “Total AFR” for 92 (82.7), “Total SEA” for 17 (14.9), “Total Americas” for 1 (0.9) “Total Europe” 1 (0.9), “Total EM for 1 (0.9) and, “Total global” for 2 (1.8) respectively.

34/45 (76%) studies used data up to and including 2020. 16/45 (36%) included data from 2021 onwards. For the purposes of this review and alignment of observations, data collection study-specific time periods (SSTPs) from publications were simplified to “pre-COVID,” “COVID-interruption,” “early-recovery,” and “late-recovery” blocks. Individual study data collection periods are detailed in Supplementary Appendix S5.

39/45 (87%) of studies were quantitative, 3/45 (7%) qualitative and 3/45 (7%) were mixed. 34/45 (76%) reported on vaccination data, 4/45 (9%) reported on measles disease outbreaks, and 7/45 (16%) used a combination of both.

### Measles Vaccination Coverage Was Severely Impacted by COVID-19

**FIGURE 2:**
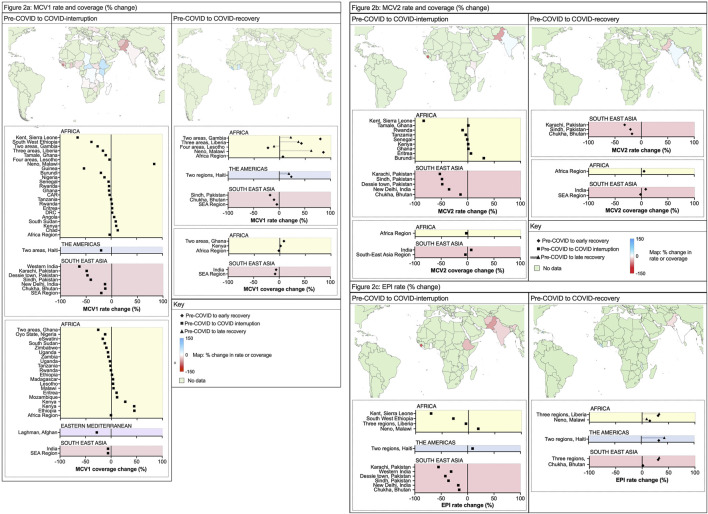
**(A)** Percentage change in measles-containing-vaccine first-dose (MCV1) vaccination rate and coverage (Low-income countries and lower-middle income countries, 2020–2023) **(B)** Percentage change in measles-containing-vaccine second-dose (MCV2) vaccination rate and coverage (Low-income countries and lower-middle income countries, 2020–2022) **(C)** Percentage change in Expanded Program on Immunisation (EPI) rate (Low-income countries and lower-middle income countries, 2020–2022).

Figure 2 summarises the reported changes in measles vaccination policy implementation from pre-COVID performance, expressed as either the percentage change in vaccination rate or the percentage change in population coverage (as used in each publication). The MCV1 rate more accurately reflected the dynamic impact from COVID-19, whereas the coverage includes an index cumulative risk of future disease outbreaks. More studies provided data on the first dose of a measles containing vaccine (MCV1). Further details on how individual studies measured rate and coverage are in Supplementary Appendix S6A including data source, and type of immunisation programme (for example, routine programmes and/or supplemental immunisation activities).

For MCV1, there was a wide range in impact from the COVID-19 pandemic; from −27.8% to +34.6% (*n* = 18 studies) and −13% to +44.4% (*n* = 19 studies) for vaccination rate and coverage respectively. LMICs reported a greater magnitude of interruption compared with LICs, with national-level data from 8/11 (73%) of LMICs that reported failures in sustained MCV1 relative to 10/22 (46%) of LICs. The worst affected countries were South Sudan (−27.8%) and Guinea (−25.5%) [19]. Two studies reported data from whole WHO regions and noted a greater loss of MCV1 in the SEA region (−20.4% rate, −6% coverage) relative to the AFR region (−2.9% rate, −1% coverage).

Several national and international studies reported an increase in MCV1 use during COVID-19 [32]. For Kenya (+44% MCV1 rate during COVID-19) this was attributed to SIAs and a new supply of measles vaccines following a stockout from November 2019 to January 2020 [51]. Bello et al reported data from 14 African countries: 7 of 14 increased MCV1 coverage in 2020 without SIAs, ranging from +1% (Ethiopia) to +27% (Kenya) [25]. Of studies that reported on local/regional data, 15 reported on MCV1 vaccination coverage changes in a total of 18 areas within countries. Here, the median change in MCV1 vaccine was −23.3% (rate) and −19.2% (coverage) from pre-COVID to COVID-interruption SSTPs. This was more severe than the overall national rate and coverage estimates. 17/18 (94%) local areas from the 15 studies reported a reduction in MCV1 rate or coverage, and this was most significant in Sierra Leone (−65.6%, rate) [26] and Western India (−63.2%, rate) [37].

### Measles Vaccination Catch-Up After the COVID-19 Pandemic

At the level of national and WHO regions, 6/41 (15%) areas assessed by 12 studies reported data on pre-COVID and early-recovery. Two studies identified further reductions in MCV1 coverage (Figure 2A) following the COVID-interruption SSTPs, both in whole WHO regions from −6% (2019–2020) to −8% (2019–2021) in the SEA region, and from −1% to −2% in the AFR region (2019–2020) [22, 23].

From studies reporting within-country (local) data, only 9/18 (50%) included data on MCV1 rate or coverage in early-recovery SSTPs. Here, the median percentage change in vaccination rate improved to +6.75% (from −23.3%). 5/9 (56%) studies reported greater MCV1 levels (rate or coverage) in early-recovery SSTP compared to pre-COVID. As before, there was wide variation with 4/9 (44%) reporting reduced MCV1 rate or coverage in early-recovery and three areas improved from their COVID-interruption performance. The changes in rate in LICs ranged widely from −65.6% to +84.2%, and for LMICs this ranged from −63.2% to −3.2%.

Recovery phase data were available on MCV1 rates for both early- and late-recovery periods for five local areas within Haiti, Lesotho, Liberia, Malawi, and the Gambia [41, 52]. 4/5 (80%) countries showed higher MCV1 vaccination rates prior to COVID-19 when compared with late-recovery. Of these four countries, three reported lower vaccination rates in late-recovery data than early-recovery SSTPs indicating difficulties in sustaining recovery momentum. Taken collectively, these data suggest many regions suffered an inability to address immunity gaps created by the pandemic and continued to have ongoing difficulties with concurrent vaccination activity.

The efficacy of a single-dose of measles vaccine can be as high as >90%, although two-doses are required for optimal protection [62]. Regarding the second dose, or MCV2 (Figure 2B), vaccination rate change from pre-COVID to COVID-interruption SSTPs in national data showed variation from −8.1% to +7.3% (n = 7 studies). Three zones reported MCV2 coverage percentage change between pre-COVID to COVID-interruption and to early-recovery: SEA region (−3%, reduced further to −5%) and AFR region (+7%, improved to +8%) [23], and India (−4%, improved to −2%) [39]. The MCV2 rate change in local data showed significant reductions (median: −48.2%, mean: −30.2%, range −83.7%–0.9%). In the early-recovery period, median MCV2 rate remained below pre-COVID levels at −18% (compared to MCV1 which recovered to +7% from −23%).

### EPI Vaccination Coverage

Two MCV doses are included in the WHO expanded programme of immunisation (EPI) schedule. Of each region reporting EPI coverage or rate, Supplementary Appendix S6A provides further information on the specific immunisations included within their reported EPI schedule. Of 11 regions with details on EPI vaccination, 10/11 (90.9%) included either measles vaccine 1 or equivalent (for example, measles-rubella 1) within this data. 6/11 (54.5%) included measles vaccine 2 or equivalent. Using within-country or local data EPI data (Figure 2C), the median rate change from COVID-interruption was −28.54%, range −70.8% to +18.6% (compared to: −23.02% MCV1; −48.2% MCV2). Local recovery in vaccination rate showed similar improvement for EPI (+8%) compared to MCV1 (+7%) between pre-COVID to early-recovery SSTPs.

The most impacted vaccines within the EPI schedules varied between studies. In a tertiary hospital setting in Pakistan, Rahman et al reported that the MCV1 and MCV2 vaccination rate was the single most affected antigen within the EPI schedule, with lowest recovery when comparing percentage change for COVID-19 interruption and early recovery with pre-COVID baseline vaccination rates (EPI vaccines −36.2% to −11.7%; MCV1 -40.9% to −17.8%; MCV2 -48.6% to −20.7%) [48]. Utazi et al [57] hypothesised that MCV1 and MCV2 showed a disproportionate reduction over other EPI vaccine antigens because they are administered later in life, as opposed to those given at birth (potentially with a healthcare provider present) or in the first few months of life. Contrarily, studies in Pakistan, Ghana and Sierra Leone identified a greater reduction in doses given at the start of the childhood schedule and attributed this as due to a reduction in births in hospitals [26, 43, 46, 48]. Dorjey et al [30] identified an overall drop of in-hospital deliveries of 16% in pandemic years in Bhutan. Kissi et al [44] reported in Ghana that, prior to COVID-19, “children delivered by traditional birth attendants outside the healthcare sector are more likely to miss immunization because their mothers become hesitant to immunize their children.” COVID-19-related disruption and fewer in-hospital births may combine to hinder EPI coverage due to a relative reduction of healthcare contact.

### Further Inequalities in Measles Vaccination Provision

Key parameters impacting measles vaccination and challenges to vaccine delivery are summarised in Tables 2, 3 respectively.

**TABLE 2:** Key vaccination parameters (Low-income countries and lower-middle income countries, 2020–2022).

GENDER						
Location (reference)	Urban or rural	Parameter	Time period			Impact
			Pre-COVID		During-COVID	
Sindh, Pakistan [47]	Both	% female vaccinated children	48.2%		47.50%	−0.7% (*p* = 0.0001), reduction in females vaccinated
Karachi, Pakistan [46]	Urban	Reduction in work force by gender			−13.7% female, −6.3% male	More significant decrease in female vaccinator work attendance
		Female child enrolment for vaccination during lockdown	120,508 (49.2%) of 245,131		11,970 (48.4%) of 24,720	Proportional decrease in females enrolled
Laghman, Afghanistan [55]	Rural	Gender differences in overall childhood vaccination	Boys 93,416		Boys 77,230	Boys −17%, girls −26%, proportional decrease in females vaccinated
			Girls 91,480		Girls 68,057	

OUTREACH PROGRAMMES						
Location (reference)	Urban or Rural	Parameter	Time period			Impact
			Pre-COVID	During‐COVID	Post-COVID	
Sindh, Pakistan [47]	Both	Average daily number of MCV1 doses to 0–23 months children (fixed; outreach)	2,444 (1,233; 1,211)	1,286 (976, 310)		- More significant decline in outreach vaccinations: MCV1 -47.4% overall (−20.8% outreach, −74.4% outreach); MCV2—48.2% (−21.2% fixed, - 73.0% outreach)
		Average daily number of MCV2 doses to 0–23 months children (fixed; outreach)	1896 (909; 986)	982 (716; 266)		
Karachi, Pakistan [46]	Urban	MCV1 doses given per day (fixed; outreach)	757 (494; 263)	387 (351; 36)	645 (478; 168)	More significant decline in outreach vaccinations, with slower recovery post-COVID than fixed vaccination sites
		MCV2 doses given per day (fixed; outreach)	560 (368; 192)	265 (242; 23)	383 (275; 108)	
Borno state, Nigeria [58] (Nomhwange)	Multi-district	Post-outbreak outreach programme—MCV1 (following 2019–20 measles outbreak)		181,634 children vaccinated. (15.4% MCV1)		Validated post-campaign coverage: 96.3% (95% CI: 93.0–98.1)
Bagmati (Dhading, Chitwan and Kathmandu) and Gandaki (Gorkha), Nepal [42]	Multi-district	MR SIA outreach following 2019–20 measles outbreak (vaccinated; targeted)		31,329; 32,150		97.4% coverage (81%–105% between districts). Early success of SIA during COVID: used new criteria to only target districts meeting specific COVID-related framework
Laghman, Afghanistan [55]	Rural	Total childhood immunisations (fixed; outreach)	184, 896 (150,185; 34,711)	145,287 (130,042; 15,245)		325 children per day missed vaccination (across all antigens and all districts)
		MR1 childhood immunisations (fixed; outreach)	9,830 (7,379; 2,451)	7,033 (6,051; 982)		Overall −28% (−18%, −60%)

URBAN AND RURAL						
Location (reference)	Parameter		Time period			Impact
			Pre-COVID	During-COVID	Post-COVID	
Sindh, Pakistan [47]	% of vaccinees from rural zone		43.4%	42.3%		Rural zone more affected (stating reduced vaccine access in rural communities)
Khyber-Pakhtunkhwa, Pakistan [48]	Urban vs. rural changes (compared to baseline before COVID-19 disruption)			−36% urban, −17% rural	−12% urban, −2% rural	Urban zone more affected (stating urban hospitals diverted to COVID care, rural hospitals continued normal care and had less COVID-19 circulation)

HOUSEHOLD TYPE						
Location (reference)	Urban or rural	Parameter	Time period			
			During-COVID			Impact
Sindh, Pakistan [47]	Both	% vaccination reduction in temporary shelters vs. non-temporary shelters	−53.8% in temporary shelters, −51.3% permanent			Greater impact on those living in temporary shelters

DELAYED VACCINES						
Location (reference)	Urban or rural	Parameter	Time period			Impact
			Pre-COVID	During-COVID	Post-COVID	
Gwalior, Madhya Pradesh, India [38]	Both	Number of vaccines delayed by > 2 weeks		3 (5.9%)		5.9% delay in MR in vaccines 01-FEB-2020 to 31-AUG-2020
Rajasthan, India [35]	Both	Odds ratio to receive timely MCV (≤9 months)	1	0.88	0.55	Odds ratio of timely vaccination reduced
		Odds ratio to receive non-timely MCV (10–12 months)	1	1.32	1.76	Odds ratio of delayed vaccination increased
Pune, Maharashtra, India [40]	Both	Total, delayed (% delayed of total) vaccination of all childhood immunisations	6,547, 1,078 (16%)	4,052, 1,089 (26.9%)	5,062, 1,165 (23%)	Increasing delayed vaccination during COVID, which worsened in 2021
Kilifi, Kenya [50]	Rural	Proportion of participants vaccinated <3 months of age-eligibility for MCV1 by year of age-eligibility for vaccination (95% CI)	2018: 78.2% (70.5, 84.7%); 2019 84.6% (76.8, 90.6%)	2020: 85.2% (81.6, 88.3%)	-	0.6% increase 2019 to 2020
Southwest Ethiopia [34]	Both	Timeliness of MCV1 vaccination within 3 months if vaccinated (95% CI)	91.9% (84.7, 96.4%)	96.4% (91.5, 96.4%)	98.2% (94.8, 99.6%)	6.3% (pre vs. post COVID)

RATE OR COVERAGE						
Location (reference)	Urban or rural	Parameter	Time period			Impact
			Pre-COVID	During-COVID	Post-COVID	
Yaounde, Cameroon [29]	Urban	Number of children receiving MMR				Significant drop in MMR vaccines from March 2020 (no numerical data provided)
Dessie town, Ethiopia [31]	Urban	MCV1 coverage	MCV1	-	400/610 (65.6%)	65.6% and 55.2% of included children had received MCV1 and MCV2 respectively
		MCV 2 coverage	MCV2	-	337/610 (55.2%)	54.6% of included children were fully vaccinated. Overall vaccination rate of all EPI declined
Pune, Maharashtra, India [40]	Both	Number of children vaccinated with MR/month	110 (est)	60 (est)	70 (est)	−35% (2019 compared to 2020)

**TABLE 3:** Vaccination challenges discussed within each article (Low-income countries and lower-middle income countries, 2020–2022).

Reason	Number of studies citing reason	Quotes from individual studies
Fear of COVID	21	“Declines in vaccine rates coincided with peaks in COVID-19 case fatality” [52]
		“Discomfort around health facility policies changing.” [54]
		“Parental reluctance to get children vaccinated out of the fear of exposing their children to infection during a vaccination session.” [47]
Re-direction of resources to COVID-19	17	“COVID-19 stay-at-home messages overwhelmed the message that the immunisation programme was to operate as normal.” [38]
		“Negative economic impacts of COVID-19, concerns that hospital will not be able to pay staff due to lost revenue.” [29]
Cold chain, infrastructure, vaccine transport	16	“Transient declines in BCG and polio vaccinations in Lesotho in October 2020 were due to stock-outs, demonstrating the pandemic’s effect on supply chains.” [52]
		“Faulty fridges impacted storage, lack of transportation and financial renumeration.” [28]
		“Disruptions in global manufacturing and supply chains, border closures, restriction on local mobility.” [47]
		“Lack of fridges in remote health centres.” [33]
		“Lack of functional fridges” [25]
Insufficient resources/resource challenges	14	“Unclear public health protocol for safe vaccine administration in a mass campaign environment.” [61]
		“2021 nationwide measles vaccination campaign postponed due to pandemic and global supply shortage.” [57]
		“Overburdened health systems, depreciation in standard delivery of immunization services in regions of Zimbabwe.” [63]
		“Reports for seven (23%) of 30 countries indicated challenges with vaccine supply” [21]
Lockdowns	14	“Immunisation of new-borns were temporarily deferred in Bhutan during nationwide lockdown periods.” [30]
		“Reduced urban vaccination because needed public transport [to access vaccination centre], which was cancelled due to COVID.” [48]
		“Ban on interstate movement reduced access to healthcare facilities.” [57]
		“Reduction in immunisation clinics *p* < 0.0001. In April immunisation services were completely suspended.” [36]
		“Lockdowns caused restricted movement.” [45]
Social distancing	13	“Parents not bringing children to hospitals due to social distancing recommendations.” [40]
Reduced/postponed outreach/mobile vaccination drives	11	“Health facilities were exempt from lock down, however immunisation outreach sessions discontinued due to fear they may spread COVID-19” [43]
		“Loss of mobile vaccination drives, loss of communication of mobile brigades, fewer vaccinators in mobile brigades.” [54]
		“Strict restrictions on movements of vaccinators to do outreach. Reduction of 79.3% (total EPI vaccinations) and 74.4% (MCV1) via outreach, compared to 32.1% (total EPI vaccinations) and 20.9% (MCV1) via outreach.” [47]
		“Cancelled SIAs—SIA was planned in 2020 in the south, postponed due to COVID-19 pandemic.” [57]
Staff absences	10	“3% decline in the public health sector staffing level.” [27]
		“Reduction in mean proportion of vaccinators attending work, with greater loss of female workers (13.7%, 95% CI: 12.9–14.5) compared to male vaccinators (6.3%, CI: 6.0–6.6).” [47]
		“Attendance of staff reduced from 91.6% to 78.7% between baseline and lockdown” [46]
		“Reduction in health workforce availability in five (45%) of 11 countries.” [21]
Vaccine hesitancy	8	“Fear of immunisation side effects.” [43]
		“Incorrect communication: link identified between child delivery by traditional birth attendants and missing childhood vaccines and identified that healthcare givers were discouraging mothers from bringing children to immunisation services.” [44]
		“Vaccine hesitancy due to COVID-19 vaccines.” [57]
		“Increasing vaccine misinformation, disinformation, and hesitancy also likely contributed to declines in some countries.” [10]
Insufficient vaccines	7	“The greatest percentage drop was seen in the administration of OPV1 which was out of stock nationally from April to July 2020.” [41]
		“A large number mentioned that stock-outs were common.” [28]
		“UNEPI (Uganda National Expanded Program on Immunization) policy of unpreserved vaccines which must be discarded at the end of the session or 6 h after reconstitution … [Sites therefore prefer to] order 5-dose [vaccine] vials rather than 10-dose vials … for fear of being blamed for wastage, and may not open vials if few children are available for vaccination.” [28]
		109’: “Some parts of the country [of Pakistan] faced a stock-out of penta vaccine (and an imminent shortage of measle’).” [47]
Travel time	‘5	“Study also revealed that long distances from the healthcare facility was a barrier to vaccination.” [28]
Lack of PPE	‘5	“Patients needed to provide own mask’s.” [54]
		“PPE not available for vaccinators for first weeks of COVID lockdown.” [47]
Public health messaging	4	“COVID-19 messaging about staying home [may have] initially overwhelmed the message that the Immunisation program was to remain operating as usual.” [38]
		“Reduced communication on existing routine health services due to heightened focus on COVID-19.” [60]
Parents unaware of EPI schedule	3	“Caregivers forgetting about the 9-month appt [for MCV].” [28]
		“Low awareness about child age-eligible for vaccination, about the side effect of vaccines and measures taken for vaccine side effects in a questionnaire taken by caregivers.” [31]
COVID-19-related misinformation	3	“WhatsApp voice-note warnings that EPI clinics would vaccinate babies with western developed COVID-19 vaccines.” [41]
		“Low awareness about transmission and prevention mechanism of COVID-19 pandemic and its fatal outcome (death) was shown among caregivers who took part in questionnaire.” [31]
Political unrest	3	“Political unrest in Oromia and Addis Ababa from 30/06/2020, led to internet shutting down due to unrest over 10 days SIA period, interrupting mass mobile messaging platforms (Telegram).' [32]
		“Rural-urban migration (more flee to urban areas due to higher insurgency in rural remote areas).” [57]
		“Challenges included security concerns, required military and civilian joint task force support.” [58]
Media messages	3	“Media propaganda about COVID-19 transmission” [34]
		“Electronic and social media under-utilised in promoting EPI vaccines.” [37]
Parents busy/other commitments	1	“16.1% of caregivers who did not take their children to EPI vaccinations stated it was due to insufficient time to take children.” [31]

Abbreviations/acronyms: EPI, expanded programme on immunisation; SIA, supplemental immunisation activity; PPE, personal protective equipment.

#### Gender

Only three studies reported on the COVID-19 impact on MCV coverage by gender [46, 47, 55]. All studies reported fewer eligible females received vaccination compared to males from the same community.

#### Outreach

Local outreach programmes were consistently more impacted than static vaccination centres and had slower recovery to pre-pandemic performance [46, 47, 55]. This was also observed in international data; Shet et al [21] reported that 44% (*n* = 12) of fixed and 86% (*n* = 25) of outreach programmes were disrupted or suspended in the AFR WHO region. 71% (*n* = 5) of fixed and 57% (*n* = 4) of outreach programmes were suspended in the SEA WHO region. A study in Karachi, Pakistan [46] identified that the worst affected areas were temporary settlements. Jain et al highlighted that vulnerable populations, specifically children in less educated, poorer, and lower caste households, were disproportionately affected in Rajasthan, India [35].

Two studies assessed measles outreach programmes initiated in response to faltering measles vaccination coverage in Nepal and Nigeria [42, 58]. Nepal used a prototype outreach programme supported by UNICEF and WHO in April 2020 which achieved 97% coverage in target districts, vaccinating almost 32,000 individuals. 105/183 (57%) of mass vaccination campaigns globally, and 31/52 (60%) of MCV-specific campaigns, were cancelled or postponed between March and December 2020 [61]. 63/105 (60%) were in the AFR or SEA regions. By December 2021, 77/472 (16%) of all campaigns planned since the beginning of the COVID-19 pandemic remained postponed or had been cancelled, the majority of which were in the WHO AFR region.

#### Vaccine Delay

Five studies commented on delayed vaccinations. In Pune, India, approximately 16% of vaccine doses were administered later than scheduled in the years before COVID-19; this proportion then increased to 27% during COVID disruption and remained at 23% in the early recovery phase [40].

Many authors sought to understand the multitude of factors that resulted in the compromise of measles policy implementation (Table 3). Common themes included fear of COVID-19, social isolation measures introduced to reduce the transmission of the disease, and critical resource limitations in the vaccine cold-chain.

### Measles Disease Outbreaks

**FIGURE 3:**
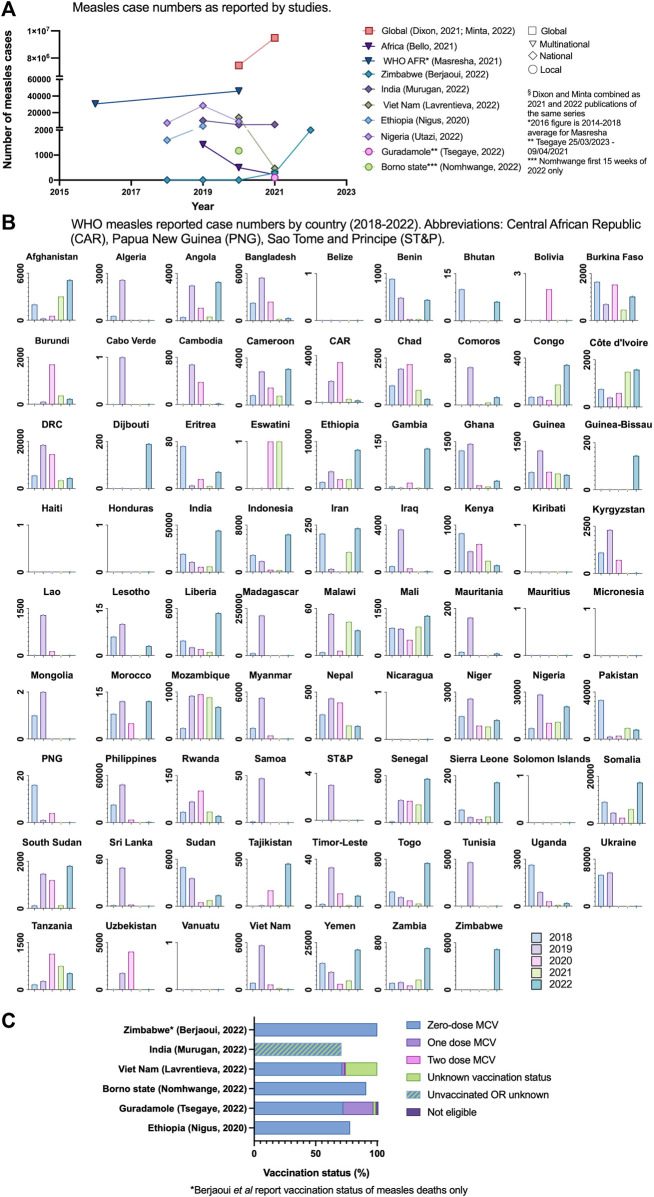
**(A)** measles case numbers as reported by studies (Low-income countries and lower-middle income countries, 2020–2022) **(B)** World Health Organisation measles reported case numbers by country 2018–2022 (World Health Organisation, 2022). **(C)** Vaccination status of confirmed measles cases or deaths (Low income countries and lower-middle income countries, 2020–2022).

11 studies documented figures on measles cases or outbreaks (Figure 3A). Two of these were local studies, six used data collected from 40 countries, four studies focussed on multinational regions (predominantly WHO regions) and two studies adopted a global perspective. Figure 3B shows the WHO measles case numbers for all LICs and LMICs from data provided from 2018 to 2022 [64].

In linked global morbidity and mortality weekly reports, Dixon et al and Minta et al reported a 26% increase in global measles cases from 2020 to 2021, with the most marked increase in the WHO Africa region of 128% (1.9 m in 2020 to 4.4 m cases in 2021) [22, 23]. Berjaoui et al were the only authors to comment on 2022 figures, documenting a single outbreak in Zimbabwe of >2000 cases and 157 deaths as of August 2022, and all deaths were in the zero-dose sub-population [63]. The total number of cases in the preceding years of 2020 and 2021 were three and 282 respectively. Overall, 6/11 (55%) of studies reported on vaccination status of confirmed measles cases or deaths during the SSTP; finding that 71%–91% of confirmed measles cases had received no doses of MCV (Figure 3C; Supplementary Appendix S7). Murugan et al reported that 71% of confirmed measles cases had received no MCV dose or had unknown vaccination status [39].

Masresha et al presented figures for measles surveillance in the WHO Africa region [20]. In 2020, 30/47 (64%) of countries met measles reporting targets (receipt of case reports from more than 80% of districts within the country). A marked reduction in suspected cases was reported in 2020; 70,242 from 323,424 in 2019. Of note, in 2019 there were large outbreaks in Madagascar, the Democratic Republic of Congo and Nigeria, attributing to a much greater number of suspected cases in 2019 compared with the average of 68,299 from 2014 to 18. Case reporting in 2020 initially reduced in April and more acutely in July and December 2020. Suspected cases in the late-recovery period had a higher blood specimen sampling rate (98% in July, September and December 2020) compared to the start of the year (32% and 41% in January 2020 and February 2020 respectively). Masresha et al proposed that below expected numbers of suspected cases reported may be due to COVID-19 related gaps in surveillance, school closures and movement restrictions. Minta et al reported similar number of specimens received in Africa in 2020 and 2021: 122,116 and 122,735 respectively and commented that the absence of a high-performing surveillance system to promptly detect cases leaves a growing proportion of susceptible children at risk from disease and outbreaks [23].

### Quality Assessments

In total, 16/49 (33%) quality assessments identified studies as good, 30/49 (61%) as fair and 3/49 (6%) as poor (see Supplementary Appendix S8 with a score of 70%–85% being fair and more than 85% being good). Of six qualitative assessments, 5/6 (83%) were reported as fair, one as good (with a score of 50%–70% being fair and >70% being good).

## Discussion

The infrastructure that underpins vaccine policy implementation is still recovering more than 2 years from the start of the pandemic. This was most noticeable and consistent in studies analysing entire WHO regions (AFR and SEA), with vaccine coverage yet to recover to pre-pandemic performance. The COVID-19 impact on measles vaccination varied more widely in local data and between individual countries. Where late-recovery time-periods were assessed, there was ongoing disruption to measles vaccination activities at a time when many of the key factors that disrupted services in the phase of high COVID-19 transmission had been resolved. Some local areas reported initial recovery to baseline immunisation activity, only to reduce again longer term, suggestive of an unsustainability in recovery programmes [52]. Measles outbreaks and deaths were substantially restricted to individuals who lacked even partial primary immunisation, and it is too early to comment on what the true, long-term impact of the COVID-19 pandemic will have on measles control.

This review builds on previous evidence highlighting the scale of disruption to immunisation programmes during the COVID-19 pandemic, utilising measles as a key consequence-indictor. A systematic review by Lassi et al highlighted immediate observations from COVID-19 in data up to September 2020, and a concurrent universal decline in vaccination coverage and four-fold increase in polio cases in polio-endemic countries [65]. Cardoso Pinto et al identified that the most severe disruption to EPI was in the first 3 months of the COVID-19 pandemic (Mar-June 2020), with ongoing disruption into late 2020 and 2021 [9]. The most recent WHO pulse survey (2022) identified that disruptions to routine immunization services increased in 2021 at the time of scale-up of COVID-19 vaccination programmes, suggesting sharp and meaningful capacity limitations in vaccine cold-chain systems [66]. Changes in MCV1 vaccination during the COVID-19 pandemic overall mirrored that of the whole EPI schedule, which supports the notion that MCV1 vaccination and measles outbreaks can act as a key indicator for overall EPI security of a population. The dominance of measles cases and deaths in unvaccinated infants shows the importance of having at least one-dose of MCV. This systematic review identified increased COVID-19 disruption to vaccination rate in national data in LMICs compared to LICs, although there did not appear to be a consistent difference between LICs and LMICs in local datasets. The 2020 and 2021 WHO pulse surveys identified that some MICs were reporting greater disruption during COVID-19 disruption than LICs [67, 68], which may be due to reporting bias given the absence of comparable data from resource-poor settings.

The pandemic resulted in a complex myriad of unprecedented factors that immediately disrupted fragile vaccine logistics in resource-poor communities. This was particularly evident from local data, which often articulated some of the challenges faced by single hospitals or areas that may be lost by reporting nationally aggregated figures. Cold-chain infrastructure limitations included stock-outs secondary to refrigerated storage space or faulty cold-chain equipment and delayed supply in multiple sub-Saharan African countries [28, 33]. Other critical infrastructure challenges were exacerbated during COVID-19, including poor road quality and transport access issues [34, 36, 60] limiting vaccine delivery to sparse rural populations. A recent UNICEF report reported that 1 in 10 children in urban zones are zero-dose (not vaccinated with any EPI vaccine) compared to 1 in 6 in rural areas [8]. Our review consistently noted a disproportionate detrimental impact on outreach programmes that target the most inaccessible and vulnerable members of the population (Figure 3C). Masresha et al [20] identified African countries with resilient vaccine infrastructure pre-pandemic, such as Senegal, Rwanda and Eritrea, maintained or recovered more rapidly to pre-COVID service levels, compared to countries such as South Sudan and Guinea.

Vaccine hesitancy has been growing globally, including to MCVs, well before the COVID-19 pandemic. A measles outbreak in Samoa in 2019 was linked to vaccine hesitancy secondary to a devastating vaccination error in 2018, in which two infants died when a multidose measles vial was reconstituted with muscle relaxant instead of water [69]. Anxiety surrounding vaccination has also increased in response to misleading information spread on social media [70]. Increasing vaccine hesitancy was cited as a specific challenge for vaccination campaigns in multiple included studies [10, 43, 44, 57].

This study identified a wide range of successfully implemented methods to improve measles vaccination coverage during a significant disease outbreak and the prioritised diversion of limited vaccine resources needed to respond to this. The first mass measles vaccination campaign during the COVID-19 pandemic, in Nepal [42], successfully triangulated surveillance data of simultaneous outbreaks (COVID-19 and measles) to ensure a directed approach based on WHO-guided principles. Subsequent post-outbreak outreach response immunisations programmes (ORIs) utilised an official bespoke and Africa-specific WHO framework for decision-making regarding implementation of mass vaccination campaigns in the context of COVID-19 [32, 58]. While an increase in COVID-19 cases was not seen in one ORI in Nigeria, operational costs increased by approximately 49% to enable COVID-19 safe methods [58]. Further locally successful methods included community engagement, including of faith leaders [42, 58, 63] and a social distance enforcer [58].

Utilising 5-dose MCV vials (over 10-dose vials) has had positive results in changing attitude and increasing vaccination [71, 72]. Vaccine vials containing higher doses per vial can result in vaccinator reluctance to open new vials unless there are enough children needing vaccines to use close to a full vial, resulting in eligible children missing out on timely MCV vaccination.

There are many vaccine technologies and infrastructure adaptations that could streamline a more robust immunisation programme, given adequate investment. These include novel delivery technologies such as micro-needles, which would reduce requirements for vaccine-related equipment [73]. Dry blood spot sampling could enhance global understanding of seroprevalence and aid direction of vaccination [74, 75]. Building resilience in vaccine cold-chain infrastructure is essential, especially in regions most affected by the evolving climate crisis.

Our study had several limitations. It was not possible to carry out a meta-analysis due to substantial methodological heterogeneity, limiting the results to descriptive analysis. The definition of pre-COVID, COVID-interruption, early-recovery and late-recovery differed within each study (Supplementary Appendix S5). These time-periods likely differed in COVID-19 intensity and COVID-19 control measures and are factors to consider when drawing conclusions regarding changes in vaccination rate or coverage. Analysis of the longer-term post-pandemic period was limited; only papers published before January 2023 were included, and the latest data reported within these studies was May 2022 [58]. There were minor differences between immunisations reported within EPI data for each region (Supplementary Appendix S6A). In most cases, statistics on EPI vaccination coverage and/or rate included measles-containing vaccines.

Studies captured immunisation data through varied methods including administrative immunisation data, research-based methods such as surveys, and coverage estimates (e.g., annual WHO-UNICEF estimates). This limits the ability to directly compare data. All above methods were included to enable assessment of a range of geographical regions and communities, including regions where formal immunisation data may not be routinely collected.

This review included studies reporting on various forms of measles vaccination programmes, including routine programmes, SIAs secondary to disrupted routine programmes, or SIAs that were otherwise planned. This was intended to provide a real-world representation of the impacts of a pandemic on mobilising immunisation campaigns that underpin vaccine coverage, with recognition that population-level herd immunity is derived from a combination of vaccination programmes. This resulted in comparison of studies reporting on routine immunisation and/or supplementary activity, where the target population and operational delivery format may differ (Supplementary Appendix S6A).

The extent to which COVID-19 disruptions impacted data obtainment is unknown. Studies did not consistently report which COVID-19-driven disruptions, or relative contributions from multiple effects, led to failure to maintain population vaccine-coverage. The focus of this paper is on all LICs or LMICs and may not capture COVID-19 effects on better resourced settings. Most of our eligible studies were from AFR WHO region, with limited information from WHO regions of the Americas (AMR) and none from Western Pacific (WPR). This may be secondary to publication bias and greater NGO presence in LICs. Data from some included studies was at risk of inaccuracies from false or under-reporting and incomplete data. Qualitative studies may have been impacted by recall bias.

### Conclusion

As the global community recovers from the effects COVID-19 pandemic, immunity gaps and limited sero-epidemiology and disease surveillance leaves millions of vulnerable children in LMICs at risk of measles. Outbreaks of measles can serve as an early predictor for the re-emergence of other vaccine preventable diseases. The post-pandemic gauntlet is to learn these lessons and establish vaccine infrastructure and systems that afford equity, efficiency, resilience, and sustainability ahead of the next health emergency [21].

## Abbreviations

AFR, Africa; CAR, Central African Republic; EPI, Expanded programme on immunisation; LIC, Low-income country; LMIC, Lower-middle-income country; MCV, Measles containing vaccine; PNG, Papua New Guinea; PRISMA, Preferred Reporting Items for Systematic Reviews and Meta-Analyses; SEA, South-east Asia; SSTP, Study-specific time periods; ST&P, Sao Tome and Principe; UNICEF, United Nations Children’s Fund; WHO, World Health Organization.
