# Supplementary material for: Measles Vaccine Coverage and Disease Outbreaks: A Systematic Review of the Early Impact of COVID-19 in Low and Lower-Middle Income Countries
Source: Int J Public Health. 2024 Apr 25;69:1606997. doi: 10.3389/ijph.2024.1606997 (PMC11079172; doi:10.3389/ijph.2024.1606997)

**APPENDICES**

**Supplementary appendix 1: PRISMA guidelines checklist**

| **Section and Topic** | **Item #** | **Checklist item** | **Location where item is reported** |
| --- | --- | --- | --- |
| **TITLE** | | |  |
| Title | 1 | Identify the report as a systematic review. | Title |
| **ABSTRACT** | | |  |
| Abstract | 2 | See the PRISMA 2020 for Abstracts checklist. | Abstract |
| **INTRODUCTION** | | |  |
| Rationale | 3 | Describe the rationale for the review in the context of existing knowledge. | Introduction, paragraphs 1-3 |
| Objectives | 4 | Provide an explicit statement of the objective(s) or question(s) the review addresses. | Introduction, paragraph 4 |
| **METHODS** | | |  |
| Eligibility criteria | 5 | Specify the inclusion and exclusion criteria for the review and how studies were grouped for the syntheses. | Methods, study selection, paragraph 1 |
| Information sources | 6 | Specify all databases, registers, websites, organisations, reference lists and other sources searched or consulted to identify studies. Specify the date when each source was last searched or consulted. | Methods, search strategy |
| Search strategy | 7 | Present the full search strategies for all databases, registers and websites, including any filters and limits used. | Methods, search strategy Supplementary appendix 2. |
| Selection process | 8 | Specify the methods used to decide whether a study met the inclusion criteria of the review, including how many reviewers screened each record and each report retrieved, whether they worked independently, and if applicable, details of automation tools used in the process. | Methods, study selection, paragraph 3 |
| Data collection process | 9 | Specify the methods used to collect data from reports, including how many reviewers collected data from each report, whether they worked independently, any processes for obtaining or confirming data from study investigators, and if applicable, details of automation tools used in the process. | Methods, Data collection, synthesis and presentation, paragraph 1 |
| Data items | 10a | List and define all outcomes for which data were sought. Specify whether all results that were compatible with each outcome domain in each study were sought (e.g. for all measures, time points, analyses), and if not, the methods used to decide which results to collect. | Methods, Data collection, synthesis and presentation, paragraph 1 |
|  | 10b | List and define all other variables for which data were sought (e.g. participant and intervention characteristics, funding sources). Describe any assumptions made about any missing or unclear information. | Methods, study selection, paragraph 1; Methods, Data collection, synthesis and presentation, |
| Study risk of bias assessment | 11 | Specify the methods used to assess risk of bias in the included studies, including details of the tool(s) used, how many reviewers assessed each study and whether they worked independently, and if applicable, details of automation tools used in the process. | Methods, quality assessment |
| Effect measures | 12 | Specify for each outcome the effect measure(s) (e.g. risk ratio, mean difference) used in the synthesis or presentation of results. | –Methods, Data collection, synthesis and presentation |
| Synthesis methods | 13a | Describe the processes used to decide which studies were eligible for each synthesis (e.g. tabulating the study intervention characteristics and comparing against the planned groups for each synthesis (item #5)). | Methods, study selection |
|  | 13b | Describe any methods required to prepare the data for presentation or synthesis, such as handling of missing summary statistics, or data conversions. | N/A |
|  | 13c | Describe any methods used to tabulate or visually display results of individual studies and syntheses. | Methods, Data collection, synthesis and presentation |
|  | 13d | Describe any methods used to synthesize results and provide a rationale for the choice(s). If meta-analysis was performed, describe the model(s), method(s) to identify the presence and extent of statistical heterogeneity, and software package(s) used. | Methods, study selection |
|  | 13e | Describe any methods used to explore possible causes of heterogeneity among study results (e.g. subgroup analysis, meta-regression). | N/A |
|  | 13f | Describe any sensitivity analyses conducted to assess robustness of the synthesized results. | N/A |
| Reporting bias assessment | 14 | Describe any methods used to assess risk of bias due to missing results in a synthesis (arising from reporting biases). | Methods, quality assessment;  Supplementary appendix 8 |
| Certainty assessment | 15 | Describe any methods used to assess certainty (or confidence) in the body of evidence for an outcome. | N/A |
| **RESULTS** | | |  |
| Study selection | 16a | Describe the results of the search and selection process, from the number of records identified in the search to the number of studies included in the review, ideally using a flow diagram. | Results, figure 1 |
|  | 16b | Cite studies that might appear to meet the inclusion criteria, but which were excluded, and explain why they were excluded. | Results, paragraph 1;  Figure 1 |
| Study characteristics | 17 | Cite each included study and present its characteristics. | Table 1 |
| Risk of bias in studies | 18 | Present assessments of risk of bias for each included study. | Results, quality assessments;  Supplementary appendix 8 |
| Results of individual studies | 19 | For all outcomes, present, for each study: (a) summary statistics for each group (where appropriate) and (b) an effect estimate and its precision (e.g. confidence/credible interval), ideally using structured tables or plots. | Results section, figure 2, table 2, figure 3 |
| Results of syntheses | 20a | For each synthesis, briefly summarise the characteristics and risk of bias among contributing studies. | Results, quality assessment;  Supplementary appendix 8 |
|  | 20b | Present results of all statistical syntheses conducted. If meta-analysis was done, present for each the summary estimate and its precision (e.g. confidence/credible interval) and measures of statistical heterogeneity. If comparing groups, describe the direction of the effect. | Nil statistical analyses |
|  | 20c | Present results of all investigations of possible causes of heterogeneity among study results. | Supplementary appendix 5 |
|  | 20d | Present results of all sensitivity analyses conducted to assess the robustness of the synthesized results. | Nil |
| Reporting biases | 21 | Present assessments of risk of bias due to missing results (arising from reporting biases) for each synthesis assessed. | Results, quality assessment;  Supplementary appendix 8 |
| Certainty of evidence | 22 | Present assessments of certainty (or confidence) in the body of evidence for each outcome assessed. | Results, quality assessment;  Supplementary appendix 8 |
| **DISCUSSION** | | |  |
| Discussion | 23a | Provide a general interpretation of the results in the context of other evidence. | Discussion paragraph 1 and 2 |
|  | 23b | Discuss any limitations of the evidence included in the review. | Discussion, paragraph 7 , 8 and 9 |
|  | 23c | Discuss any limitations of the review processes used. | Discussion, paragraph 7, 8 and 9 |
|  | 23d | Discuss implications of the results for practice, policy, and future research. | Discussion, paragraphs 4,5 and 6 |
| **OTHER INFORMATION** | | |  |
| Registration and protocol | 24a | Provide registration information for the review, including register name and registration number, or state that the review was not registered. | –Methods, registration |
|  | 24b | Indicate where the review protocol can be accessed, or state that a protocol was not prepared. | –Methods, registration |
|  | 24c | Describe and explain any amendments to information provided at registration or in the protocol. | N/A |
| Support | 25 | Describe sources of financial or non-financial support for the review, and the role of the funders or sponsors in the review. | Detailed in submission portal |
| Competing interests | 26 | Declare any competing interests of review authors. | Detailed in submission portal |
| Availability of data, code and other materials | 27 | Report which of the following are publicly available and where they can be found: template data collection forms; data extracted from included studies; data used for all analyses; analytic code; any other materials used in the review. | Data extraction tables available on request |

**Supplementary appendix 2: Search strategy**

**OVID MEDLINE(r) and EMBASE:**

1. *Exp Measles/*
2. *Measles.mp.*
3. *MCV*.mp.*
4. *MMR.mp*
5. *MR.mp*
6. *1 or 2 or 3 or 4 or 5*
7. *Vaccin*.mp*
8. *Immuni*.mp*
9. *Inoculat*.mp*
10. *7 or 8 or 9*
11. *Program*.mp*
12. *EPI.mp*
13. *(Essential AND program* AND immuni?ation).mp*
14. *SIA.mp*
15. *(Supplementary AND immuni?ation AND activit*).mp*
16. *Schedule.mp*
17. *Cover*.mp*
18. *PIRI.mp*
19. *(Periodic and Intensification and Routine and Immuni?ation).mp.*
20. *11 or 12 or 13 or 14 or 15 or 16 or 17 or 18 or 19*
21. *6 AND 10 AND 20*
22. *Year limit: 2020-2023*
23. *Humans only limit*

**PubMed:**

1. *‘Explode: Measles/’ OR measles OR MCV1 OR MCV2 or MCV OR MMR OR MR*
2. *Vaccin* OR Immuni* OR Inoculat**
3. *Program* OR EPI OR (Essential AND program* AND immuni?ation) OR SIA OR (Supplementary AND immuni?ation AND activit*) OR Schedule OR Cover* OR (Periodic AND Intensification AND Routine AND Immuni?ation) OR* PIRI
4. #1 AND #2 AND #3
5. *Humans only*
6. *2020-2023*

**Supplementary appendix 3:** inclusion of WHO region data based on proportion of lower-middle and low-income countries (LMICs), as of February 2023.

|  | **No. of countries in WHO region** | **No. of countries LIC in 2020** | **No. of countries LMIC in 2020** | **% of countries within region with =>75% LIC/LMIC in 2020** |
| --- | --- | --- | --- | --- |
| ***1 African Region (AFR)*** | 47 | 22 | 18 | ***85.10638298*** |
| 2 Region of the Americas (AMR) | 35 | 0 | 5 | 14.28571429 |
| ***3 South-East Asian Region (SEAR)*** | 11 | 1 | 8 | ***81.81818182*** |
| 4 European Region (EUR) | 53 | 0 | 4 | 7.547169811 |
| 5 Eastern Mediterranean Region (EMR) | 21 | 5 | 8 | 61.9047619 |
| 6 Western Pacific Region (WPR) | 37 | 0 | 10 | 27.02702703 |
| *Where studies analysed whole WHO regions, data from WHO regions were included were >=75% of countries within the region were classed as LMIC or LIC during 2020.* | | | | |

**Supplementary appendix 4:** Full study summaries

|  |  |  |  | **Bias assessment** | |
| --- | --- | --- | --- | --- | --- |
|  | **First author** | **Year** | **Summary of study** | **Quantitative** | **Qualitative** |
| 1 | Osei *et al* | 2022 | This study assessed rural regions in Gambia for the impact of childhood attendance and vaccination during the COVID-19 pandemic. They identified three months of EPI interruption, including a 15.5% reduction in MCV1 coverage. A positive recovery (79.5%) was seen compared with pre-COVID-19 baseline. | Good | - |
| 2 | Connolly *et al* | 2022 | Study assessing childhood immunisation during COVID-19 in Haiti, Lesotho, Liberia and Malawi. It identified that measles vaccination rates in Haiti, Lesotho and Liberia had a statistically significant decline in March to August 2020 with an upward trend at the end of this period. | Good | - |
| 3 | Atim *et al* | 2021 | A secondary analysis of Ugandan government-led portals assessing COVID-19 impact on childhood and maternal health. It demonstrated a 6.8% reduction in measles vaccine coverage (88% July 2018 to June 2019, compared to 82% July 2019 to June 2020). | Good | - |
| 4 | Bhadoria *et al* | 2021 | Cross sectional study of Jaya Arogya Group of Hospitals (JAH), a tertiary hospital in India. It demonstrated delayed vaccination for Measles-Rubella 1 in 5.9% of children. The number of vaccinations given increased at the end of the monitoring period. | Fair | - |
| 5 | Jain *et al* | 2021 | Study assessing disruption to childhood immunisation due to COVID-19 in Rajasthan, India. Children due MCV1 between March and May 2020 were significantly less likely to receive it at or before 9 months and more likely to receive it at an older age. Catch up efforts did not offset immunisation lapses. | Fair | - |
| 6 | Bimpong *et al* | 2021 | Study analysing the impact of COVID-19 on childhood immunisation in northern Ghana. They identified a decline in measles vaccine during the pandemic of 10.5%. This was the smallest decline of any EPI vaccine. | Good | Fair |
| 7 | Powelson *et al* | 2022 | Study in Mozambique assessing childhood immunisation drop-outs. 19% of children in this region start but do not complete vaccination schedule. Barriers to vaccination that they identified include: immunisation burden on mother, poor trust in health care services, concerns about side effects, healthcare power imbalances and COVID-19 creating barriers. | - | Good |
| 9 | Kissi *et al* | 2022 | Study analysing impact of COVID-19 disruption on childhood immunisation, including MCV, in Accra, Ghana. Measles vaccination rates in the Weike Gwabe municipality of Ghana dropped from 64.7% (2019) to 38.8% (2020). | Fair | - |
| 10 | Walekhwa *et al* | 2022 | Study assessing gaps in measles vaccination coverage in rural Uganda. They identified many key barriers in rural Uganda, including: vaccine stock outs leading to supply disruption in already underserved child populations, minimal vaccine storage and delays in the national supply system and staff burn out. | - | Fair |
| 11 | Chelo *et al* | 2021 | Study analysing impact of COVID-19 on attendance and routine vaccinations at a paediatric referral hospital in Cameroon. They identified that paediatric consultations dropped in April and May 2020 by 52% and 34% respectively. As a result, vaccine demand including MMR dropped significantly. | Good | - |
| 12 | Babatunde *et al* | 2022 | Study assessing impact of COVID-19 restrictions on routine immunisations in Oyo State, Nigeria. Study identified a drop in routine immunisation rates. measles dropped sharply from 77.0% to 64.6% respectively. None of the planned fixed and outreach sessions occurred. | Poor | - |
| 13 | Dorjey *et al* | 2022 | Study assessing impact of COVID-19 pandemic period on the maternal and child health unit at a general hospital in Bhutan, through comparison of 2018 to 2019 period with 2020 to 2021 period. Limited data specifically on routine immunisation coverage in postnatal period. | Good | - |
| 14 | Miretu *et al* | 2021 | Qualitative study via interviews of caregivers to children who underwent EPI vaccination either before or during COVID lockdown in Dessie town, Ethiopia. Age-eligible vaccination rate was 79.2% pre-COVID. The study identified factors associated with full or partial vaccination. They identified that good knowledge level on COVID-19 shows statistically significant association with full vaccination status for routine childhood immunisations. | Fair | Fair |
| 15 | Chandir *et al* | 2020 | Analysis of immunisation records from real-time provincial electronic immunisation registry in Sindh, Pakistan, for September 23, 2019, to July 11, 2020, and compared the 6 months before lockdown with the COVID lockdown period. They identified that one of every two children in Sindh province missed their routine vaccinations during first COVID lockdown. | Good | - |
| 16 | Rahman *et al* | 2021 | Assessment of the impact of the COVID-19 pandemic on a health centre in Pakistan, comparing 5 months before lockdown (November 2019 to March 2020), 5 months from the beginning of the first lockdown (April 2020 to August 2020) and 5 months after lockdown (September 2020 to January 2021). Study included assessment of the impact of vaccination rates in rural vs urban zones, and identified greater impact in urban zones. Assessed impacts on individual EPI vaccine types, identifying biggest drop in immunisation rates in MCV2. | Fair | - |
| 17 | Shet *et al* | 2022 | Paper reviewing the impact of SARS-CoV-2 on routine immunisation services within 170 countries and territories. It identified global disruption and highlighted concerns of VPD outbreaks in future. Identified early signs of recovery as of the end of 2020. Paper studied all six WHO regions, data therefore extracted from AFR and SEA region. | Fair | - |
| 18 | Ho *et al* | 2022 | Study assessing impact of SARS-CoV-2 disease on mass vaccination campaigns (including MCV) internationally. They assessed these at 4 time points: May 2020, December 2020, May 2021 and December 2021. Study highlighted significant disruption both to outbreak responses and preventive campaigns. | Fair | - |
| 19 | Chandir *et al* | 2020 | Study which used EIRs to identify changes in vaccination rates of routine immunisations in lockdown period in comparison to same time period in 2019 in Karachi, Pakistan. Identified significant discrepancies in vaccination based on wealth status, with concerns of lower immunisation rates in areas of temporary housing. | Good | - |
| 20 | Buonsenso *et al* | 2020 | Retrospective cross-sectional study assessing vaccinations at a rural health post in Sierra Leone (estimated population coverage 5000 people). They compared vaccination during 01/03/2020 to 26/04/2020 with the same time period in 2019. | Fair | - |
| 21 | Nigus *et al* | 2020 | Secondary analysis of an MCV SIA carried out during COVID-19 pandemic in July 2020 in Ethiopia. Results show the SIA reached 102.8% administrative coverage nationwide, with 78% of 1123 woredas attaining target of 95% coverage. | Fair | - |
| 22 | Utazi *et al* | 2022 | Bayesian geostatistical modelling to map the routine coverage of first doses of DTP1 and MCV1 with corresponding zero-dose estimates in Nigeria. They assessed areas at 1x1km resolution and district level with geospatial datasets. Further mapping of MCV1 coverage in various settings, including: before and after 2019 measles vaccination campaign in association with zero-dose children (particularly focused in Northern states where there are more zero-dose children), reported cases of measles in 2018 to 2020. They identified similar high- and low-coverage zones for DTP1 and MCV1, with higher case numbers for measles in zero-dose zones. | Good | - |
| 23 | Berjaoui *et al* | 2022 | A narrative review of online data assessing the impact of the measles outbreak in Zimbabwe August 2022, with information on reasons for outbreak and subsequent management, including government mass vaccination campaign for children in and near areas of outbreak. | - | Fair |
| 24 | Tsegaye *et al* | 2022 | Retrospective case-control study of a measles outbreak which occurred in the Guradamole Woreda of Bale Zone, Ethiopia. Study assessed associated reasons for cotnracting measles and highlighted possible factors leading to measles outbreak in 2021 after disruption to SIAs during COVID. Guradamole Woreda had an MVC coverage of 75% in 2020. | Good | - |
| 25 | Nomhwange *et al* | 2022 | Retrospective review of the ORI (outbreak response immunisation) during COVID-19 pandemic to measles outbreaks in 6 local government areas of Borno state, Nigeria. Utilised the WHO decision making framework to decide where/whether to implement an ORI in that region. | Fair | - |
| 26 | Bose *et al* | 2022 | Study analysing when the National Immunisation Programme (NIP) of Nepal implemented a measles outbreak response immunisation (ORI) campaign in 2020. It identified districts based on a novel multi-dimensional logic framework to inform decisions with support and advice from WHO and UNICEF to carry out ORI during COVID-19 large-scale transmission in Nepal. They saw a successful reduction of measles incidence as a result of ORI, with consideration of where to vaccinate based on multiple factors, including where COVID transmission low to reduce impact on COVID rates. This was additional to an MR SIA campaign which was interrupted due to COVID-19 lockdown measures. | Good | - |
| 27 | Murugan *et al* | 2022 | Morbidity mortality weekly report (MMWR) from 2005 to 2021 in India. Identified interesting change that between 2019 to 2021, measles cases went from greatest proportion seen in zero-dose children, to greatest proportion seen in children with two or more doses. | Fair | - |
| 28 | Dixon *et al* | 2021 | Morbidity mortality weekly report (MMWR) from 2019 to 2020 of global measles vaccination and incidence rates, assessing current progressing towards measles elimination. Study identified significant decline in global MCV1 coverage in 2020. | Fair | - |
| 29 | Minta *et al* | 2022 | Morbidity mortality weekly report (MMWR) from 2000 to 2021 of global measles vaccination and incidence rates, assessing current progressing towards measles elimination. Study highlighted lowest MCV1 coverage in 2021 globally since 2008. Overall measles incidence shown to decrease in comparison to 2019. | Fair | - |
| 30 | Lavrentieva *et al* | 2022 | Assessment of acute measles circulation through measure of IgM levels in individuals throughout Viet Nam from January 2020 to March 2021. Study noted active measles spreading in early 2020, with reduction during 2020. They hypothesis that this could have been due to COVID lockdown measures. Measles spread mainly amongst unvaccinated (71.58%) or those unaware of vaccination status (25.48%). 71% <3 years. | Poor | - |
| 31 | Muhoza *et al* | 2021 | Study reports global, regional and national vaccination coverage estimates and trends as of 2020. It monitors vaccination coverage, including unvaccinated and under-vaccinated children, for vaccines including DTP and MCV1. | Fair | - |
| 32 | Rachlin *et al* | 2022 | Study reports global, regional and national vaccination coverage estimates and trends as of 2021. It monitors vaccination coverage, including unvaccinated and under-vaccinated children, for vaccines including DTP, Pol3, MCV1. | Fair | - |
| 33 | Ludvigsson *et al* | 2022 | Systematic literature review of Ukrainian healthcare and the health of Ukrainian children, spanning from 2010 to 2022. Paper is not specific to impact of COVID on immunisation, but it includes data from 2019 compared to 2020 for overall vaccination rates for EPI, including MCV. | Fair | - |
| 34 | Abid *et al* | 2022 | Assessed a single province in rural Afghanistan for measles vaccination rates and other EPI vaccinations in April to July 2019 compared to April to July 2020. | Fair | - |
| 35 | Pawar *et al* | 2022 | Study assessed overall number of children vaccinated for EPI programme (including MCV) in a tertiary centre in Pune, India, comparing 2019 to 2021. | Good | - |
| 36 | Masresha *et al* | 2021 | Analysed measles case-based surveillance and lab databases in the WHO Africa region (including individual country data) between 2014 and 2020 to assess impact of COVID-19 pandemic on measles surveillance. It found reduction in suspected measles cases being reported beginning from April 2020. Regarding sampling, it identified lower total number of blood specimens received, of which greater proportion were positive for measles. | Fair | - |
| 37 | Lucinde *et al* | 2023 | Analysed vaccination rates (including MCV) before, during and after COVID-19 disruption in set of children who became eligible for vaccination either before, during year 1 or during year 2 of pandemic (3 retrospective cohorts) in Kilifi, one of Kenya's poorest counties. They identified that the COVID19 pandemic did not adversely affect vaccine coverage in this area, with statistically significant higher rates of vaccination in pandemic year 2. | Fair | - |
| 38 | Masresha *et al* | 2020 | Summary of impact of COVID-19 pandemic on EPI schedules (including MCV) through comparison of January to March 2020 and April to June 2020 in 15 African countries. Data was collected from administrative reporting data from routine immunisation programmes to analyse trends. Main comparisons were of DPT and MCV vaccines. | Good | - |
| 39 | Baloch A *et al* | 2021 | A retrospective cross sectional study of 1169 health facilities in 22 districts in Sindh, Pakistan. This paper demonstrated a decrease in scheduled MCV1 vaccines of 17.3% and 54.3% in March 2020 and April 2020 respectively, when compared with January and February 2020 | Fair | - |
| 40 | Barasa *et al* | 2021 | This mixed qualitative and quantitative retrospective national Kenyan study demonstrated a declining monthly trend in MCV coverage between Jan-19 and Feb-20 (pre-COVID) of 2% per month. In March 2020 measles vaccine coverage increased by 44.44%, From April-20 to Nov-20 MCV coverage declined at a rate of 0.77% per month. Kenya had relatively a relatively moderate COVID-19 restrictions. The increase in MCV coverage in Mar-20 is due to increased outreach following a stockout Nov-19 to Jan-20. | Fair | Fair |
| 41 | Bello *et al* | 2021 | A multinational cross-sectional study of 19 countries in the east and southern Africa region, 14 of which are LIC or LMIC. They report that 6 (32%) of countries have increasing MCV coverage and 13 (68% of countries have decreasing MCV1 coverage in Jan-Aug 20 compared with Jan-Aug 19. This conclusion was not consistent with the presented data on MCV1, which demonstrates 7 countries with increased coverage and 12 with decreased coverage. | Fair | - |
| 42 | Kassie *et al* | 2021 | Cross sectional study in South West Ethiopia demonstrating that number of MCV1 vaccines given dropped by 38.54% in March-June 2020 compared with the same period of 2019. | Good | - |
| 43 | Khan *et al* | 2021 | A single centre retrospective observational study in New Delhi, India, that identified reductions in MCV1, MCV2 and total vaccines given of -13.1%, -35.3% and -17.7% respectively during COVID-19 compared with prior to COVID-19. This study comments on subsequent emergences of measles cases in the provinces served by this vaccination centre. | Fair | - |
| 44 | Kinikar *et al* | 2021 | A single-centre, retrospective study in Western India with prospective data analysis. It demonstrated a decline in weekly average of MCV1 and overall vaccines given of -63.2% and -31.2% respectively. | Fair | - |
| 45 | Sato *et al* | 2021 | Cross-sectional national study in Nigeria using regression analysis to demonstrate a reduction in overall and MCV1 vaccines given in April and May 2020, followed by a recovery from June 2020. | Good | - |
| 46 | Wanyana *et al* | 2021 | A national cross-sectional study examining the impact of COVID-19 on Maternal and Child Health (MCH) services in Rwanda. It demonstrated reduced utilisation of basic MCH services during March and April 2020. Measles vaccination utilisation was not significantly affected. | Fair | - |
| **Acronyms:** EIR, electronic immunisation record; VPD, vaccine-preventable diseases; EPI, Expanded Programme of Immunisation; SIA, supplementary immunisation activity. | | | | | |
|  |  |  |  |  |  |

**Supplementary appendix 5:** Data collection timings per study


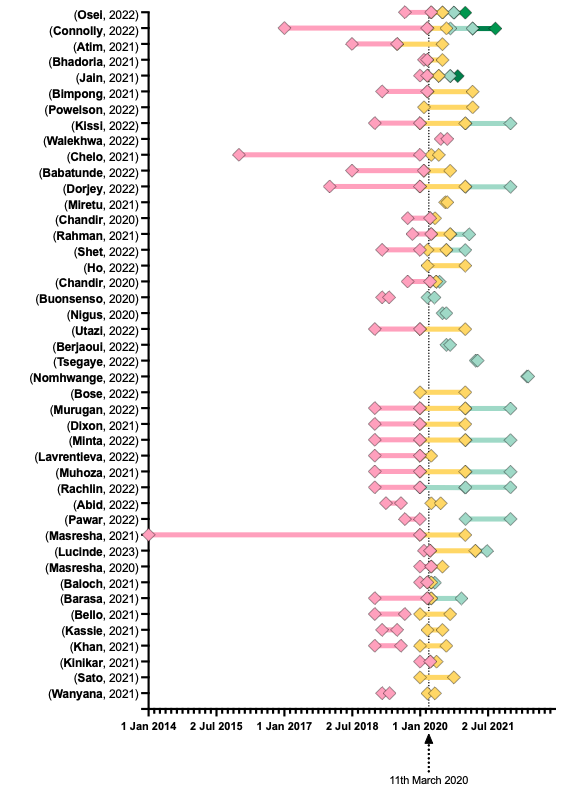


11^th^ March 2020: date WHO pronounced COVID-19 to be a pandemic.

**Key of study-specific time-periods (SSTPs):**

1. Pink: pre-COVID
2. Yellow: mid-COVID
3. Light green: early-recovery
4. Dark green: late-recovery

**Supplementary appendix 6a:** Unit of measurement used by papers reporting vaccination rate or coverage.

| Paper | Measurement | Unit of measurement | Data source | Routine immunisation (RI) / supplementary immunisation activity (SIA) | EPI vaccines included:* |
| --- | --- | --- | --- | --- | --- |
| Osei *et al*, 2022 | Rate | Number of vaccines given per month | Basse and Fuladu West Health &  Demographic Surveillance Systems | RI | N/A |
| Connolly *et al*, 2022 | Rate | Number of vaccines given per month | Health management information systems (collected at facility level) | RI | BCG, OPV0, OPV1, OPV2, OPV3, Penta1, Penta2, Penta3, Penta4, PCV1, PCV2, PC3, Rota1, Rota2, MCV1 |
| Atim *et al*, 2021 | Coverage | % vaccine coverage | Secondary data  obtained from Ugandan government-led portals | RI | N/A |
| Bimpong *et al*, 2021 | Rate | Number of vaccines given per month | Electronic records from the Child Welfare Clinic at the Tamale Teaching hospital | RI | N/A |
| Kissi *et al*, 2022 | Coverage | % vaccine coverage | District Health Information Management  System (DHIMS) | RI | N/A |
| Babatunde *et al*, 2022 | Coverage | % vaccine coverage | The monitoring and evaluation unit  of Oyo State Primary Health Care Board | RI | N/A |
| Dorjey *et al*, 2022 | Rate | Number of children vaccinated in one hospital per year | Medical records of clinic attendees | RI | BCG, HepB0, OPV0, OPV1, OPV2, OPV3, Penta1, Penta2, Penta3, MR1, MR2, IPV |
| Chandir *et al*, 2020 | Rate | Number of children aged 0-12 months vaccinated per day | Provincial Electronic  Immunization Registry | RI | BCG, OPV0, OPV1, OPV2, OPV3, Penta1, Penta2, Penta3, PCV1, PCV2, PCV3, Rota1, Rota2, IPV, Meas1, Meas2 |
| Rahman *et al*, 2021 | Rate | Average monthly routine childhood immunisation visits per vaccine type | Hospital Immunization  Registry | RI | BCG, Meas1, Meas2, Rota1, Rota2, Penta1, Penta2, Penta3, Polio+PCV1, Polio+PCV2, Polio+PCV3 |
| Shet *et al*, 2022 | Rate | Mean relative difference in vaccine administrations | WHO collected data from regional offices | RI | N/A |
| Chandir *et al*, 2020 | Rate | Number of doses given per day | Provincial Electronic  Immunization Registry | RI | BCG, OPV0, OPV1, OPV2, OPV3, Penta1, Penta2, Penta3, PCV1, PCV2, PCV3, Rota1, Rota2, IPV, Meas1, Meas2 |
| Buonsenso *et al*, 2020 | Rate | Number of children vaccinated in one health post per 12 month period | Health facility routine activity reporting forms | RI | BCG, OPV0, OPV1,OPV2, OPV3, Penta1, Penta2, Penta3, PCV1, PCV2, PCV3, Rota1, Rota2, IPV, IPTI1, IPTI2, IPTI3, Meas1, Meas2, YF |
| Nigus *et al*, 2020 | Coverage | % vaccine coverage | Ethiopia Demographic Health Survey | RI and SIA** | N/A |
| Murugan *et al*, 2022 | Coverage | % vaccine coverage | WHO and UNICEF Joint Reporting Form aggregations | RI and SIA | N/A |
| Dixon *et al*, 2021 | Coverage | % vaccine coverage | WHO and UNICEF estimates from administrative records | RI and SIA | N/A |
| Minta *et al*, 2022 | Coverage | % vaccine coverage | WHO and UNICEF estimates from administrative records | RI and SIA | N/A |
| Abid *et al*, 2022 | Coverage | % vaccine coverage | Secondary data from the Ministry of Health | RI | BCG, HepB, IPV, Meas1/MR1, OPV0, OPV1, OPV2, OPV3, OPV4, PVC1, PCV2, PCV3, Penta1, Penta2, Penta3, Rota1, Rota2 |
| Lucinde *et al,* 2023 | Coverage | % vaccine coverage | Vaccine coverage survey from stratified random sample within the Kilifi Health and Demographic Surveillance System | RI | N/A |
| Masresha *et al*, 2020 | Rate | Monthly number of children vaccinated nationally | Administrative reporting data | RI | N/A |
| Barasa *et al*, 2021 | Coverage | % vaccine coverage | Secondary data from the Kenya Health Information System database | RI*** | N/A |
| Bello *et al*, 2021 | Coverage | % vaccine coverage | Routine month administrative immunisation coverage data | RI | N/A |
| Kassie *et al*, 2021 | Rate | Number of vaccines given per quarter in Governmental Health Facilities in South West Ethiopia | Review of hospital and health centre records by nurses or midwives | RI | Total newborn vaccinated count (vaccines not specified) |
| Khan *et al*, 2021 | Rate | % change in number of vaccines given in Jan-Jul 2020 compared with the same period in 2019 in an immunization centre in New Delhi | Local immunisation records | RI | BCG, OPV0, OPV1, OPV2, OPV3, HepB, Penta1, Penta2, Penta3, fIPV1, fIPV2, OPV, MR1, MR2, DPT booster, OPV booster, DPT booster 2, TT1, TT2 |
| Kinikar *et al*, 2021 | Rate | Average number of vaccines given per week at a single tertiary care centre | Computerised vaccination records from the vaccination centre | RI | BCG, OPV0, OPV1, HepB, Penta1, Rota1, IPV, MR1, DPT booster |
| Wanyana *et al*, 2021 | Rate | Number of vaccines given nationally in March and April 2020 compared with March and April 2019 | Rwanda Health Management information system | RI | BCG, OPV0, OPV1, OPV2, OPV3, IPV, Penta1, Penta2, Penta3, PCV1, PCV2, PCV2, Rota1, Rota2, MR1, MR2 |

*BCG: Bacillus Calmette-Guérin, OPV: Oral Polio Vaccine, Penta: Pentavalent vaccine,, PCV: Pneumococcal conjugate vaccine, Rota1: Rotavirus vaccine, Meas: Measles vaccine, MR: Measles-Rubella vaccine, HepB: Hepatitis-B vaccine, IPV: Inactivated polio vaccine, IPTI: Intermittent preventative treatment in infants, fIPV: fractional dose inactivated polio vaccine, DPT: Diphtheria-Tetanus-Pertussis Vaccine, TT: Tetanus Toxoid

**Study data is of total administrative coverage. Paper specifically comments on SIAs occurring within this time.

*** Based on routine immunisation data. However, following a stock-out, health workers reached out to unvaccinated children to encourage update of the measles vaccine.

**Supplementary appendix 6b:** Manuscript figure 2b including the paper author.


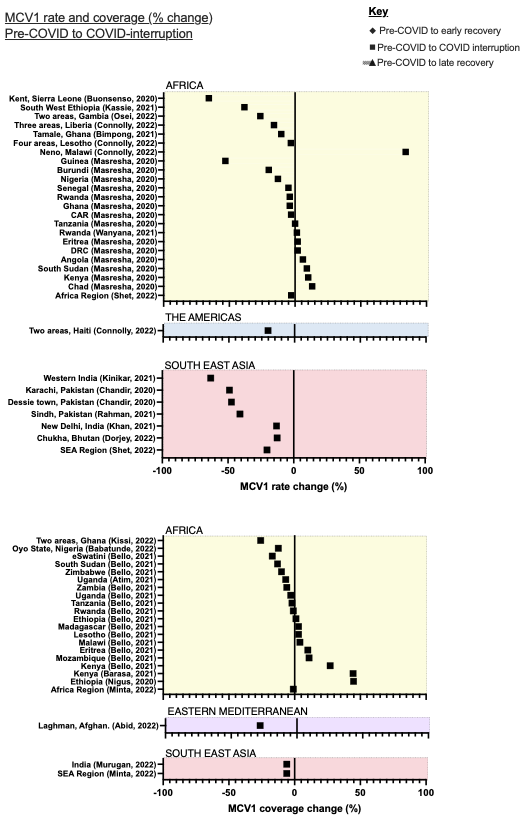

**Supplementary appendix 7:** vaccination status of reported measles cases and deaths (where available)

| **Paper (deaths/cases)** | **Total number** | **Vaccination status (%)** | | | | | |
| --- | --- | --- | --- | --- | --- | --- | --- |
|  |  | **Zero-dose** | **One-dose** | **Two-dose** | **Unknown** | **Zero-dose or unknown** | **Not eligible** |
| **Nigus *et al*, 2020: Ethiopia (cases)** | 1846 | 78 |  |  |  |  |  |
| **Tsegaye *et al*l, 2022, Guradamole (cases)** | 98 | 72.45 | 24.49 | 0 | 2.04 |  | 4.08 |
| **Nomhwange, 2022, Borno state (cases)** | 1176 | 91 |  |  |  |  |  |
| **Lavrentieva *et al*, 2022; Viet Nam (cases)** | 468 | 71.58 | 2.14 | 0.64 | 25.64 |  |  |
| **Murugan *et al*, 2022; India (cases)** | 46223 |  |  |  |  | 71 |  |
| **Berjaoui *et al*, 2022, Zimbabwe (deaths)** | 157 | 100 |  |  |  |  |  |

**Supplementary appendix 8: bias assessment results**

**8a) Quantitative bias assessment results (n=43)**


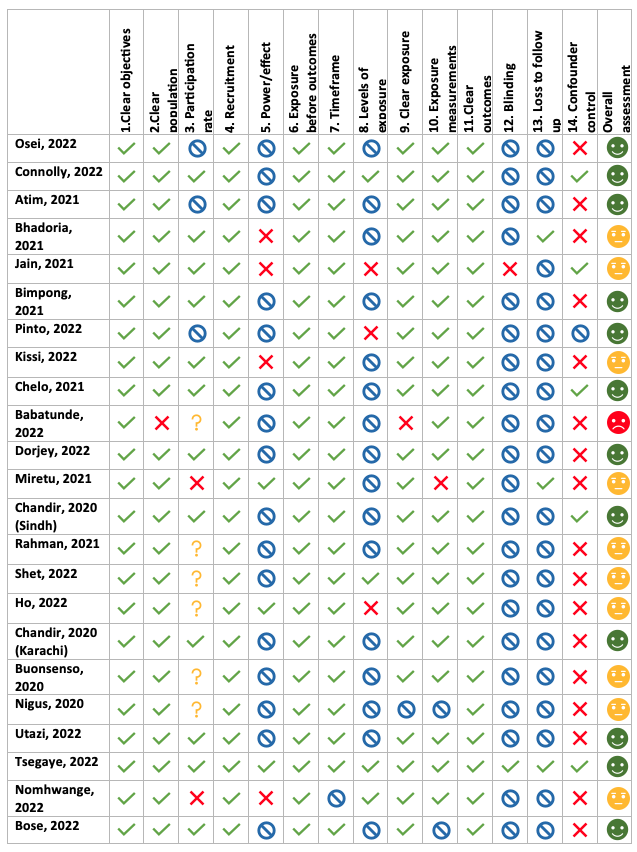

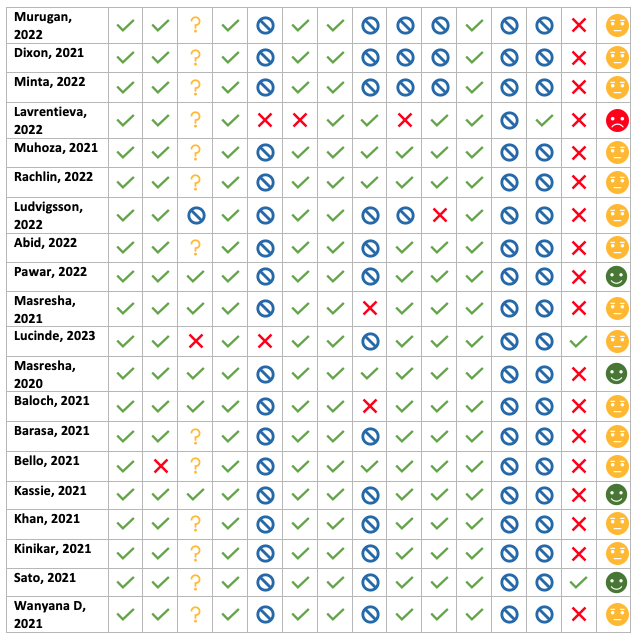


**8b) Qualitative bias assessments (n=6):**


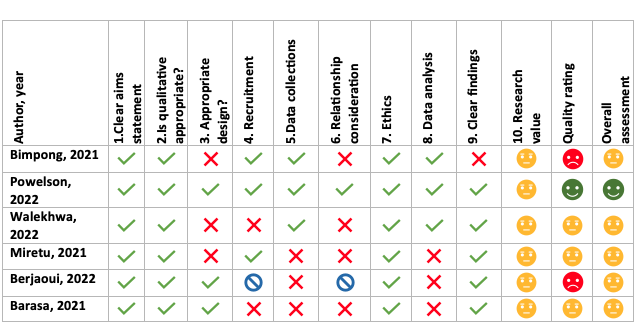

Supplement: Supplementary file 1 [file DataSheet1.docx]
